# Clinical Features of Severe Wasp Sting Patients with Dominantly Toxic Reaction: Analysis of 1091 Cases

**DOI:** 10.1371/journal.pone.0083164

**Published:** 2013-12-31

**Authors:** Cuihong Xie, Shabei Xu, Fengfei Ding, Minjie Xie, Jiagao Lv, Jihua Yao, Dengji Pan, Qian Sun, Chenchen Liu, Tie Chen, Shusheng Li, Wei Wang

**Affiliations:** 1 Department of Neurology, Tongji Hospital, Tongji Medical College, Huazhong University of Science and Technology, Wuhan, Hubei, China; 2 Department of Emergency, Tongji Hospital, Tongji Medical College, Huazhong University of Science and Technology, Wuhan, China; 3 Department of Cardiology, Tongji Hospital, Tongji Medical College, Huazhong University of Science and Technology, Wuhan, China; 4 Division of Clinical Immunology, Tongji Hospital, Tongji Medical College, Huazhong University of Science and Technology, Wuhan, PR China; University Heart Center Freiburg, Germany

## Abstract

**Background:**

Massive wasp stings have been greatly underestimated and have not been systematically studied. The aim of this study was to identify the clinical features and treatment strategies of severe wasp stings.

**Methods and Findings:**

A multicenter retrospective study was undertaken in 35 hospitals and medical centers including 12 tertiary care hospitals and 23 secondary care hospitals in the Hubei Province, China. The detailed clinical data of 1091 hospitalized wasp sting patients were investigated. Over three-fourths (76.9%) of the cases had 10 or more stings and the in-hospital mortality of patients was 5.1%. Forty-eight patients died of organ injury following toxic reactions to the stings, whereas six died from anaphylactic shock. The in-hospital mortality in patients with >10 stings was higher than that of ≤10 stings (5.2% vs. 1.0%, p = 0.02). Acute kidney injury (AKI) was seen in 21.0% patients and most patients required blood purification therapy. Rhabdomyolysis was seen in 24.1% patients, hemolysis in 19.2% patients, liver injury in 30.1% patients, and coagulopathy in 22.5% patients. Regression analysis revealed that high creatinine level, shock, oliguria, and anemia were risk factors for death. Blood purification therapy was beneficial for patients with ≥20 stings and delayed hospital admission of patients (≥4 hours after sting).

**Conclusions:**

In China, most patients with multiple wasp stings presented with toxic reactions and multiple organ dysfunction caused by the venom rather than an anaphylactic reaction. AKI is the prominent clinical manifestation of wasp stings with toxic reaction. High creatinine levels, shock, oliguria, and anemia were risk factors for death.

## Introduction

Wasps are critically important in natural bio-control and in preserving an ecological balance in agriculture. Wasp stings are not uncommon worldwide. In developed countries, almost all patients who have suffered from one or a few stings have had allergic reactions of varying degrees [1]. Therefore treatment is focused on anti-anaphylaxis and desensitization [2], [3]. However, in comparison with the occasional incidence reported previously, wasp sting induced injuries have been occurring more frequently in recent years in China, which has caused considerable mortality among victims and become an increasingly serious public health problem [4]–[6]. Some reports from other developing countries, including India, Vietnam, Thailand, Malaysia, and Nepal, also indicate that victims attacked by wasps in swarms probably undergo a life-threatening toxic reaction in response to the venom [7]–[11]. Although wasp stings have become a serious public health problem, they has been greatly underestimated and have received little attention. Some reports with small sample sizes have shown that wasp stings might lead to multiple organ dysfunctions [7], [8]. However, there is no systematic analysis on the clinical features, treatment strategies, and prognosis of severe wasp sting patients with large sample sizes.

Hubei is a large province with a population of over 60 million, located in the central part of China, which has a subtropical monsoonal climate with distinct contrast between the eastern plain and the western mountainous area. In the present study, the clinical data of hospitalized wasp sting patients from 2009–2011 in the Hubei Province, China, were investigated. The results provided a novel insight into the epidemiology and clinical characteristics of wasp sting and helped develop strategies of prevention and treatment.

## Materials and Methods

### Clinical data collection and analysis

Between 2004 and 2011, the annual number of patients with wasp stings admitted to 35 hospitals and medical centers including 12 tertiary care hospitals and 23 secondary care hospitals in the Hubei Province, China, were calculated. Among them, the cases admitted between 2009 and 2011 were enrolled with detailed data and this part of the study is retrospective. From Nov. 2011 on, all hospitalized patients with wasp stings from these 35 hospitals were included in a small prospective study to test for the serum venom-specific IgE and lymphocyte subsets and various inflammation mediators. There were 25 consecutive cases involved until the end of 2011. The diagnosis of wasp stings was based on clinical history and findings on physical examinations. A standard wasp sting management protocol was followed for the emergent management [12]. Detailed history was recorded and clinical examinations and investigations were performed to provide complete clinical characteristics. Not all clinical data from the laboratory analyses or overall patient evolution were available, resulting in the number of observations for calculating means to be less than 1091 in some variables. When this occurred, the corresponding number of observations was given. The study was approved by the Ethics Committee of Tongji Medical College, Huazhong University of Science and Technology. Eligible patients were given a copy of written informed consent and those who signed the informed consents were recruited into this study. For the minors/children participants, written informed consents were signed by the guardians on the behalf of them.

### Clinical characteristics

We documented the following information: presence of hematuria, hypotension or oliguria/anuria upon admission, number of stings, time between sting and hospitalization, duration of oliguria/anuria and other clinical manifestations including vomiting, hematemesis, arrhythmia, heart failure, pneumonic edema, consciousness, and other complications. We also documented duration of hospital stay, in-hospital mortality, and cause of death.

### Laboratory examination

The related laboratory examinations were obtained soon after the patients were admitted. Using these laboratory findings combined with clinical manifestations, the following diagnoses could be made: hemolysis: anemia with elevated free hemoglobin in blood; rhabdomyolysis: blood serum CK ten times higher than normal and elevated free myohemoglobin in blood; liver injury: elevated ALT; kidney injury: elevated Cr; coagulopathy: PT>17.5 s or APTT>55.0 s; anaphylactic shock: hypotension with allergic symptoms such as wheezing, angioedema and urticaria; non-anaphylactic shock: only hypotension without allergic symptoms.

### Treatment

All patients were treated with glucocorticoids and antihistamines. Those with serious allergic reactions received additional epinephrine treatment. Blood purification therapy was provided for patients with one of following symptoms: (1) multiple organs dysfunction syndrome (MODS); (2) anuria or oliguria for more than two days; (3) acute pulmonary edema; (4) severe hyperkalemia: potassium >6.5 mmol/L; (5) severe metabolic acidosis, pH<7.2; (6) apparent soy sauce-colored urine associated with daily serum BUN raised >14.3 mmol/L, and elevated Scr >177.0 umol/L. All patients received supportive symptomatic treatments.

### Measurement of venom-specific IgE assays and flow cytometry analysis

Blood samples were collected from 25 multiple sting patients within 14 days of the sting. The venom-specific IgE and lymphocyte subsets and various inflammation mediators were tested.

The quantitative determination of total serum IgE levels was performed with chemiluminescent immunoassay (CLIA) on the UniCel DXI 800 immunoassay system (Beckman Coulter). The quantitative determinations of venom-specific serum IgE levels were performed with Immuno CAP specific IgE fluoroenzyme immunoassay on the Phadia 250 instruments. Commercially available assay for specific serum IgE antibodies (Immuno CAP, Phadia, Uppsala, Sweden) were performed for the following 7 types of venom. They are for the venom of the white-faced hornet, yellow jacket, paper wasp, yellow hornet, European hornet, European paper wasp, bumblebee. Levels of specific IgE antibody were graded by 6 classes, >0.7 kU/L were considered positive (Class≥2).

Direct immunofluorescent labeled whole blood hemolysis method with flow cytometry (BD FACSCalibur) were applied to test lymphocyte subsets and the following inflammation mediators: total T lymphocytes, total B lymphocytes, auxiliary/leading T lymphocytes, inhibitory/cytotoxic T lymphocytes, and regulatory T lymphocytes (natural regulatory T lymphocytes, induction of regulatory T lymphocytes and interleukin-2 (IL-2), interleukin-4 (IL-4), interleukin-6 (IL-6), interleukin-10 (IL-10), tumor necrosis factor-α (TNF-α), interferon-γ (IFN-γ), interleukin-17α (IL-17α).

### Statistical analysis

A wasp sting survey was performed using Epidata 3.0 software. The data are expressed as a number (± standard deviation [SD]) and in total percentages for the 1091 patients or for the total number on which the variable was measured. The means and ranges for each value are also reported. Data were analyzed using SPSS software version 11.5. Multivariate binary logistic stepwise regression analysis was performed when applicable. The following independent variables were included in multiple regression analyses, including age, gender, admission time, number of sting wounds, time between sting and hospitalization, number of organs failed on admission, shock, anuria, anemia, serum phosphocreatine, serum creatinine, serum aminotransferase levels, clotting time, whether to receive blood purification, type and duration of blood purification and hospital level. A p-value of less than 0.05 for 95% confidence was set and used as a cut-off point to examine the statistical association between the variables and mortality. Multivariate binary logistic stepwise regression analysis was employed to evaluate factors associated with in-hospital mortality. Sting number groups were compared (≤10 stings group versus >10 stings group) using the Chi-square for categorical variables. For all statistical tests, α = 0.05.

## Results

### Clinical data

Between January 2004 and December 2011, 2167 patients with severe wasp stings were admitted to 35 hospitals in the Hubei province. The number of cases increased annually from 123 to 611 with a large jump of reported cases in 2011 (2.7 times that of 2010; data not shown). From 2009 to 2011, 1091 patients with severe wasp stings were admitted and we studied these cases in detail.

The majority of patients (963 cases, 88.3%) were adults; with the mean age of 42.5±19.9 (range: 0.3–87.0) years. Most patients were stung in the head and/or upper limbs and many had multiple (up to 200) stings. The majority of patients were male and of the 1091 patients, 867 cases (84.7%) were rural. The mean length of time between wasp sting and hospitalization was 10.7 hours (range: 0.1–288.0 h; Table 1).

**Table 1:** Clinical characteristics of wasp sting patients.

Age (years)	42.5 (0.3–87.0)
Child(%)^1^	128 (11.7)
Adult (%)^2^	963 (88.7)
Sex (M∶F)	620∶471
Rural residence (%)	897 (84.7)
Time elapse from bite to hospital (hours)	10.7 (0.1–288.0)
Duration of hospital admission (days)	3.8 (0.5–47.0)
Renal injury (%)	183 (21.0)
Liver injury (%)	230 (30.1)
Rhabdomyolysis (%)	75 (24.1)
Hemolysis (%)	177 (19.2)
Anuria/oliguria (%)	83 (7.7)
Hemoglobinuria (%)	110 (10.2)
Hypotension (%)	49 (4.5)
Coagulopathy (%)	88 (22. 5)
Pulmonary edema (%)	84 (7.7)
Dialysis (%)	119 (11.0)
Death (%)	54 (5.1)

^1^ <18 years of age.

^2^≥18 years of age.

### Clinical manifestations

All patients showed local inflammatory reaction (redness, swelling, hot, and pain) after the stings. Dizziness, nausea, and vomiting were the most common symptoms observed in 800 patients (73.3%). As shown in Table 1, hypotension was found in 49 patients on admission (4.5%). Gross hematuria or dark-red colored urine was detected in 110 cases (10.1%). Eighty-three patients presented with anuria or oliguria (7.6%). Loss of consciousness was found in 52 patients on admission (4.8%). Convulsion was present in 14 cases during the hospitalization; 72 cases had arrhythmia (6.6%), and 17 patients developed acute respiratory distress syndrome (ARDS) (1.6%). Altogether, 251 patients (23.0%) suffered from two or more organs dysfunctions. The length of stay in the hospital was 3.8 days (0.5–47.0 days).

### Presence of shock and mortality

As shown in Table 1, 49 patients (4.495%) presented with hypotension on admission, including 24 (22.0%) who were diagnosed with anaphylactic shock and 25 (22.9%) diagnosed with non-anaphylactic shock. Fifty-four patients died and the mortality of hospitalized patients was 5.1%. Twenty-seven patients (50.0%) died of MODS, 17 patients (31.5%) died of shock soon after admission including 6 (11.1%) of anaphylactic shock and 11 (20.4%) of non-anaphylactic shock. Other causes included hyperkalemia (9.3%), massive hemorrhage (5. 6%) and cardiac arrhythmia (3.7%).

### Incidence of acute kidney injury (AKI)

21.0% of patients (183/870) exhibited varying degrees of AKI (Table 1), and of these, 119 (11.0%) received hemodialysis. The average CK level was much higher in patients with AKI than that in non-AKI patients (14170.3 IU/L vs. 3658.5 IU/L). Nine patients with AKI had CK levels <195 IU/L. Most survivors had complete recovery of renal function, although 12 patients had impaired renal function when observed 6 months post-release. The mean age of these 12 patients was higher than that of the completely recovered patients (56.5 vs. 45.6 years).

### Incidence of rhabdomyolysis and hemolysis

Elevated CK was found in 53.7% patients (167/311) including 24.1% (75/311) diagnosed with rhabdomyolysis (Table 1); 58 rhabdomyolysis patients developed AKI which was higher than that in the total patients (79. 5% vs. 21.0%, p<0.001); 19.2% (177/923) patients presented with hemolysis and 64 of these patients developed AKI, which was also higher than that in the total patients (36.2% vs. 21.0%, p<0.001).

### Venom hepatotoxicity

Transaminase and bilirubin levels were measured in 765 patients (Table 2); 230 (30.1%) patients had ALT levels >40 IU/L and 343 (44.8%) had AST levels >40 IU/L implying liver injury. However, the transaminase levels especially AST, are not accurate indexes due to the concurrent rhabdomyolysis. Elevated total bilirubin levels were seen in 360 (47.1%) patients with an average level of 55.3 µmol/L.

**Table 2:** Laboratory data of patients.

	No. of	No. of cases with abnormal	Mean of abnormal data	Range of
Laboratory test(normal)	cases	data (%)	(±SD)	abnormal data
Hematology				
Hemoglobin (120–160 g/L)	923	177(19.2)	98.1(±17.8)	19.0–119.0
Platelets (100–300*10^9^/L)	923	85(9.2)	73.3(±21.3)	20.0–99.0
Leukocytes (4–10*10^9^/L)	923	635(68.8)	17.5(±7.0)	10.1–51.2
Neutrophils (50–75%)	923	719(77.9)	87.5 (±5.3)	75.1–97.4
Blood chemistry				
Creatinine (52–104 µmol/L)	870	183(21.0)	273.0 (±283.9)	104.3–1829.7
Blood urea nitrogen (2.3–7.1 mmol/L)	870	241(27.7)	14.4(±10.2)	7.1–53.3
Alanine aminotransferase (0–40 IU/L)	765	230(30.1)	399.4(±786.1)	40.1–6995.0
Aspartate aminotransferase (0–40 IU/L)	765	343(44.8)	769.5 (±1894.8)	40.1–14862.0
Serum total bilirubin (3.4–17.1 mmol/L)	765	360(47.1)	55.3 (±66.3)	17.2–479.5
Serum conjugated bilirubin (0–6.8 mmol/L)	765	269(35.2)	24.2 (±36.4)	6.9–265.4
Creatine kinase (0–195 IU/L)	311	164(52.7)	6536.3(±12031.6)	198.0–74844.0
Lactate dehydrogenase (0–235 IU/L)	296	152(51.4)	2084.6(±2859.5)	246.0–14098.0
Glycemia (3.9–6.1 mmol/L)	555	250(45.1)	10.8(±3.4)	7.8–30.1
Blood coagulation test				
Prothrombin time (11–14.5 s)	392	135(34.4)	18.1(±6.3)	14.6–62.9
Activated partial prothrombin time (32–45 s)	392	148(37.8)	94.6(±52.0)	45.2–290.4
Urine test				
Qualitative proteinuria(negtive)	596	90(15.1)		
Qualitative hematuria(negtive)	596	146(24.5)		
Flow cytometry analysis				
IL-6 (5 pg/ml)	25	17(68.0)	28.1(±37. 6)	5.6–149.5
Regulatory T lymphocyte (CD3+CD4+CD25+CD27low+)(4.59–7.81%)	25	21(84.0)	1.6(±1.0)	0.2–3.4
Natural regulatory T lymphocyte (CD45RA+CD3+CD4+CD25+CD27low+)(2.07–4.55%)	25	21(84.0)	0.3(±0.3)	0–0.9

### Coagulation disorder

Coagulation disorders were commonly seen and appeared to be related to venom dose; 34.4% patients (135/392) had PT >14.5 s and 37.8% patients (148/392) had APTT >45.0 s (Table 2). The mean PT and APTT values were 18.1 s and 94.6 s, respectively. 22.5% patients (88/392) had PT >17.5 s or APTT >55.0 s and were diagnosed with coagulopathy.

### Correlation of the number of stings with the severity of outcome

The severity of symptoms appeared to be dependent on the number of stings, with 76.9% cases (657/854) suffering from more than 10 stings. As shown in table 3, the overall incidence of AKI was higher in >10 stings group (21.0%, p = 0.039) than those in the ≤10 stings group (12.9%). It was also observed with the elevated CK levels (56. 3% vs. 28.6%, p = 0.002). Patients with >10 stings had a higher occurrence of liver injury and coagulation disorder (elevated ALT 31.8% vs. 13.5%, p<0.001; elevated PT 26.1% vs. 14.1%, p = 0.026; elevated APTT 36.0% vs. 21.8%, p = 0.017) compared to those with ≤10 stings. The percentage of patients with anemia was higher in the >10 stings group (21.1% vs. 9.0%, p = 0.001). There was also a significant difference in in-hospital mortality between the two groups (1.0% vs. 5.2%, p = 0.02).

**Table 3:** Relationship between number of wasp stings and clinical presentation/outcome.

No. of envenomation	≤10	>10	P value
No. of patients	197	657	
Deaths (%)	2/196(1.0%)	33/638(5.2%)	0.02
Elevated creatinine (%)	16/124(12.9%)	116/552(21.0%)	0.039
Elevated BUN (%)	21/124(16.9%)	128/552(23.2%)	0.129
Descended HGB (%)	13/144(9.0%)	122/578(21.1%)	0.001
Elevated ALT (%)	14/104(13.5%)	156/490(31.8%)	<0.001
Elevated AST (%)	27/104(26.0%)	223/490(45.5%)	<0.001
Elevated CK (%)	10/35(28.6%)	119/212(56.1%)	0.002
Elevated LDH (%)	12/31(38.7%)	102/197(51. 8%)	0.176
Extended PT (%)	11/78(14.1%)	82/314(26.1%)	0.026
Extended APTT (%)	17/78(21.8%)	113/314(36.0%)	0.017

### Multivariate classification Logistic regression analysis

Multivariate classification logistic regression analysis revealed that high Scr levels (p = 0.0003, OR: 8.761 [95% CI: 2.697–28.457]), shock (p = 0.0044, OR: 7.07 [95% CI: 1.844–27.386]), oliguria (p = 0.0216, OR: 4.053 [95% CI:1.228–13.374]), and anemia (p = 0.0139,OR : 3.674 [95% CI:1.303–10.365]) are risk factors of in-hospital mortality. Although age (>60 yrs) had no relationship with in-hospital mortality, it did weakly correlate to observed renal dysfunction (p = 0.0265, OR: 2.107 [95% CI: 1.302–3.411]). Blood purification therapy was effective for patients with more than 20 stings and admitted more than 4 hours after sting (p = 0.0107, OR: 0.066 [95% CI: 0.006–0.722]).

### Flow Cytometric Analysis

Flow cytometric analysis revealed that the serum IL-6 level of severe cases increased significantly compared to controls, whereas regulatory T lymphocytes, especially natural regulatory T lymphocytes significantly declined (Table 2).

### Venom-specific IgE tests

Among the 25 seriously injured wasp sting patients, 16 were venom-specific IgE positive to at least 1 of 7 common wasp venom (class ≥2) that were thought to result in allergic reactions. As shown in Table 4, none of them suffered anaphylactic shock, or cardiac arrest. Only 1 of the sIgE(+) patients presented with gasp for breath. All patients complained of nausea and vomiting, tiredness and headache. Among the 16 sIgE(+) patients, 13 and 10 had oliguria and hemoglobinuria, respectively; 14 had elevated levels of Cr/BUN level and 14 had hepatic dysfunction; 12 had elevated CK levels. Meanwhile, all of the 9 sIgE(−) patients presented with positive features as mentioned above. The Chi square test did not reveal a significant difference between the sIgE(+) and sIgE(−) groups.

**Table 4:** Comparison of clinical features of venom-specific IgE(+) and venom-specific IgE(−) patients.

	Stings			Anaphylactic shock	Apnea	Nausea and vomiting	Oliguria	Hemoglobinuria	Cr/BUN level elevation	Hepatic dysfunction	CK level elevation
	10–20	20–50	>50								
sIgE(+) patients	37.5%(6/16)	56.3%(9/16)	6.3%(1/16)	*	6.3%(1/16)	100%(16/16)	81.3%(13/16)	62.5%(10/16)	87.5%(14/16)	87.5%(14/16)	75.0%(12/16)
sIgE(−) patients	22.2%(2/9)	66.7%(6/9)	11.1%(1/9)	*	*	100%(9/9)	100%(9/9)	100%(9/9)	100%(9/9)	100%(9/9)	100%(9/9)

* None of sIgE(+) or sIgE(−) patients present with this clinical feature.

## Discussion

Wasp sting events have occurred world-wide, especially in developing countries with swarm attack [13]–[15]. In comparison with the occasional incidence in previous reports, our investigation revealed that wasp stings have been frequent in recent years in China, which has caused considerable mortality among victims and become an increasingly serious public health problem [16], [17]. Some reports from other developing countries such as India, Vietnam, Thailand, Malaysia, and Nepal with small sample sizes indicate wasp attacks in swarms. However, there is no systematic analysis on the clinical features, treatment, and prognosis of severe wasp sting patients with large sample sizes. Here, we systematically analyzed the clinical data of 1091 hospitalized wasp sting patients during 2009–2011 in the Hubei Province. Hubei is a large province of China with a population of over 60 million. Thirty-five hospitals and medical centers participated in our study including 12 tertiary care hospitals and 23 secondary care hospitals, which could reflect the actual situation in the Hubei Province.

The major clinical characteristics of these patients are toxic reactions, and the wasp venom toxicity is attributed to hemolytic, myotoxic, neurotoxic, vasodilatory, nephrotoxic and hepatotoxic enzymes [18]. Signs and symptoms in our patients included shock, AKI, rhabdomyolysis, hemolysis, liver dysfunction and coagulopathy. Fatal stings usually occur in the head or neck, and death typically results from hypotension, laryngeal edema, or bronchial constriction within 1 hour. Less commonly, death occurs from the toxic effects of massive envenomation involving hundreds of stings [19]. However, in our study of all the patients who died during the hospitalization, 48 died of MODS, non-anaphylactic shock or other complications due to severe intoxication and only 6 died of anaphylactic shock. Deaths occurred more commonly from organ injury due to toxic reaction in this study. We found that 24 patients presented with anaphylactic shock and 25 with non-anaphylactic shock on admission. The cases with non-anaphylactic shock were much more serious and more difficult to treat than the cases presenting with anaphylactic shock. The mortality in the non-anaphylactic group was higher than that in the anaphylactic shock group (44.0% vs. 25.0%). A direct vasodilatory effect of wasp venom, particularly at high doses may be responsible for the non-anaphylactic shock. The rate of anaphylaxis was the same as previously reported [20], but the toxic reaction was more severe in our study. Among the 1091 cases, 23.0% from dysfunction of two or more organs, and rhabdomyolysis was observed in 24.1% patients during the hospitalization, which was caused by toxic reaction of the venom. These data also imply that toxic reactions due to massive envenomation were more common and serious than the allergic reaction.

According to the reports by Muller et al, and Ring and Messmer et al [21], [22], systemic anaphylactic reactions to wasp venom was graded into 4 classes, ranging from generalized urticaria and itching, to anaphylactic shock, cardiac arrest and apnea. Among the 25 patients we tested for venom specific IgE, none suffered anaphylactic shock or cardiac arrest. Only one of the sIgE (+) patients presented with shortness ofgasp for breath, and the majority of sIgE positive patients had the same clinical features as the sIgE negative ones who only suffered from toxic reactions. It has been suggested that most patients suffered from toxic reactions with or without allergic reactions and toxic reactions were the dominant presentation in case of multiple stings.

AKI is the most prominent clinical manifestation of wasp stings resulting in toxic reactions. AKI was observed in 21.0% of our patients. Most patients required blood purification therapy. 79.5% of patients diagnosed with rhabdomyolysis developed AKI, thereby establishing rhabdomyolysis as the main cause of AKI. The rate of AKI in hemolysis patients was also higher, suggesting that hemolysis is an associated mechanism for AKI. Previously, studies have shown that AKI due to wasp stings was secondary to pigment-induced acute tubular necrosis [23]. We also suspect a direct toxic effect from the venom on the renal tubules, particularly in those lacking elevated CK levels.

The severity of clinical manifestation is related to the number of stings. We found that the levels of almost all the laboratory tests were elevated, including Cr, ALT, CK, extended PT, APTT and descended HGB, which were higher in patients with ≥10 stings than those in patients with <10 stings. The in-hospital mortality in the ≥10 stings group was 5 times higher than that in <10 stings group, which was statistically significant. This indicates that the severity of clinical features and the prognosis depend on the number of stings in patients with toxic reactions.

The mechanisms underlying wasp sting injury may comprise the direct toxic effect of venom and immune inflammatory reaction to venom composition, both of which can lead to organ failure [24]. The level of cytokine IL-6 in serum increased, whereas regulatory T lymphocytes decreased significantly in patients in the acute phase, suggesting the induction of an immune-inflammatory reaction. Release of large amounts of inflammatory mediators may be attributable to multiple organ injury. We found that blood purification therapy can improve the prognosis of patients with ≥20 stings and delay hospital admission of patients (≥4 hours after sting). In developed countries, most wasp sting patients suffered only a single sting; as a result, anaphylactic reaction is the main clinical features. It is rare for patients to develop renal failure and MODS. Consequently, blood purification therapy was not considered in previous treatment strategies for wasp sting. In contrast, in China, most patients were attacked by a crowd of wasps, resulting in severe hemolysis and rhabdomyolysis, which greatly injures the kidney. Therefore, blood purification therapy was often applied in patients with renal failure and MODS. It is well known that blood purification therapy can remove not only the free myohemoglobin and hemoglobin due to rhabdomyolysis and hemolysis but also venom toxins and inflammatory factors, thus attenuating organ damage [25], [26]. However, the definite efficacy of blood purification therapy needs to be confirmed in prospective randomized clinical trials in the future.

Our study has several limitations. Although a standard wasp sting management protocol was followed for the emergency management, some clinical characteristics were not recorded completely, as the main part of our study was retrospective. Some patients were discharged early due to financial problems. Hence, the mortality was probably underestimated. A prospective design is needed in future studies. The efficacy of blood purification therapy should be appreciated with caution since it was not rigorously evaluated by a randomized controlled trial.

To our knowledge, this study is the biggest study on the grossly underreported problem of massive wasp stings. Different from what has been seen in developed countries, most patients stung by wasps with multiple stings suffered from toxic reactions and multiple organ dysfunctions caused by the venom rather than an anaphylactic reaction. AKI is the most common clinical manifestation of wasp stings that result in a toxic reaction. High creatinine level, shock, oliguria, and anemia were risk factors for death.
